# Study for the validation of the FeetMe^®^ integrated sensor insole system compared to GAITRite^®^ system to assess gait characteristics in patients with multiple sclerosis

**DOI:** 10.1371/journal.pone.0272596

**Published:** 2023-02-09

**Authors:** Anabel Granja Domínguez, Raúl Romero Sevilla, Aurora Alemán, Carmen Durán, Anja Hochsprung, Guillermo Navarro, Cristina Páramo, Ana Venegas, Ana Lladonosa, Guillermo Izquierdo Ayuso

**Affiliations:** 1 Departamento de Neurología, Fundación para el Desarrollo de la Investigación y Asistencia de Enfermedades Neurológicas y Afines Crónicas (DINAC), Castilleja de la Cuesta, Sevilla, Spain; 2 Departamento de Neurología, Hospital Vithas Nisa, Unidad de Investigación y Tratamiento de la Esclerosis Múltiple, Sevilla, Spain; 3 Neurociencias, Novartis Farmacéutica, S.A., Barcelona, Spain; Universita degli Studi di Napoli Federico II, ITALY

## Abstract

**Objective:**

To determine the concordance and statistical precision in gait velocity in people with multiple sclerosis (pwMS), measured with FeetMe^®^ (insoles with pressure and motion sensors) compared with GAITRite^®^ (classic reference system of gait analysis) in the timed 25-Feet Walk test (T25WT).

**Methods:**

This observational, cross-sectional, prospective, single center study was conducted between September-2018 and April-2019 in pwMS aged 18–55 years, with Expanded Disability Status Scale (EDSS) 0–6.5 and relapse free ≥30 days at baseline. Primary endpoint was gait velocity. Secondary endpoints were ambulation time, cadence, and stride length assessment, while the correlation between gait variables and the clinical parameters of MS subjects was assessed as an exploratory endpoint.

**Results:**

A total of 207 MS subjects were enrolled, of whom, 205 were considered in primary analysis. Most subjects were women (66.8%) and had relapsing-remitting MS (RRMS) (82.9%), with overall mean (standard deviation [SD]) age of 41.5 (8.0) year and EDSS 3.1 (2.0). There was a statistically significant (p<0.0001) and strong agreement (intra-class correlation coefficient (ICC) >0.830) in gait velocity, ambulation time and cadence assessment between FeetMe^®^ and GAITRite^®^.

**Conclusions:**

Agreement between devices was strong (ICC≥0.800). FeetMe^®^ is the first validated wearable medical device that allows gait monitoring in MS subjects, being potentially able to assess disease activity, progression, and treatment response.

## Introduction

Multiple sclerosis (MS) is an inflammatory, demyelinating, and neurodegenerative disease of the central nervous system that results in episodic decline of neurologic functions. It is one of the primary causes of non-traumatic disability in younger adults [1]. The Global Burden of Disease study 2016 estimated that 2.22 million people worldwide were suffering from MS, corresponding to a prevalence of approximately 30 cases per 100,000 population [2]. According to prevalence studies that have been conducted in Spain, the rate varies from 47.7–79/100,000 population-years [3,4].

In people with MS (pwMS), gait disorder is a hallmark feature that significantly impairs functional status, employment, and quality-of-life [5]. Gait disorder is characterized by multifactorial symptomatology, including weakness of lower extremities, spasticity, and postural instability due to cerebellar or vestibular dysfunction, proprioceptive sensory abnormality, vision loss, oscillopsia or diplopia [6].

Gait analysis contributes significantly to monitoring the evolution and progression of the disability in MS patients. Many types of disability measures are used for gait analysis, including clinician-assessed rating scales, patient self-report questionnaires, and performance tests. The Kurtzke’s Expanded Disability Status Scale (EDSS) [7] is a disease-specific scale that has become the gold standard for characterizing disability levels and determining disability progression in patients with MS. However, several methodological difficulties are associated with EDSS, including use of ordinal scale (0 to 10), subjectivity in certain areas (e.g., bowel and bladder function), non-specific to minor changes, inability to evaluate fall risk or gait speed, and diagnostic inaccuracy [8–11].

In addition to conventional scales, a range of semi-subjective instruments are used to assess gait disorder. The timed 25-foot walk test (T25WT) measures gait speed; but variations can exist due to instructions given for walking (such as brisk walk or walk in a comfortable speed) which may impact the consistency of the test results. Besides, the examiner cannot record fall risk, gait deviations, gait variability, gait initiation, patient’s ability to adjust gait in turns, and fatigability in the T25WT [12]. Similar limitations are inherent in other gait measurement tools like the 2-minute walk test (2MWT) or 6-minute walk test (6MWT) that are currently being used in different neurologic conditions but are yet to establish their validity in MS patients [13].

In contrast to the semi-subjective methods, qualitative gait analysis is based on the use of different wearable (e.g., inertial units, accelerometers, and depth cameras) and non-wearable devices (e.g., optical motion capture systems, force platforms, and instrumented walkways)and digital tools (such as accelerometer in smartphones and smartwatches) [14,15]. GAITRite^®^ is a sensor embedded walkway mat which is considered as the gold standard device to objectively measure different spatiotemporal parameters of gait (including functional ambulatory profile [FAP] score, velocity, cadence, ambulation time, step length, and single and double support time) [16–18]. Nevertheless, use of GAITRite^®^ is restricted in routine clinical practice due to cost, limited availability, lack of space in hospitals, need for trained personnel, and time spent on gait assessment, monitoring, and computing. Moreover, the device captures information only at a given time point in the clinical setting, depending on the patient’s situation at that time and does not assess gait capacity and performance in real-time and real-world [17,19,20].

These limitations hinder the effective evaluation of patients with gait disorders. Thus, there is a need of a device that can record different qualitative and quantitative gait parameters, seamlessly integrated even on an out-patient basis, and is easy to access and used by both physicians and patient. Better measurement of the disability progression and acute exacerbations will be an additional benefit. FeetMe^®^ is a shoe insole sensor device that allows a comprehensive and objective assessment of gait alterations in clinic and real-world settings [20]. It consists of integrated pressure and motion sensors (gyroscope, and accelerometer) to collect a wide range of clinically relevant step-by-step gait parameters (such as velocity, cadence, ambulation time, and stride length) that are not even quantified in EDSS and T25WT [21]. Despite substantial research in the field of designing wearable insole sensors and validating their clinical accuracy, certain gaps still need to be addressed *via* well-designed, high-quality real-world studies. Hence, this study was conducted to determine the concordance and statistical precision in gait velocity in study subjects with MS, measured with a device of insoles sensors (FeetMe^®^) compared with the classic reference system for analysis of gait (GAITRite^®^), in the T25WT.

## Methods

### Study objectives

The primary objective of this study was to determine the concordance and statistical precision in gait velocity (cm/sec) in study subjects with MS, measured with an insoles device that incorporates a system of pressure and motion sensors (FeetMe^®^) compared with the classic reference system of analysis of gait (GAITRite^®^) in the T25WT (7.62 meters). The secondary objectives included determination of the ambulation time (sec), gait cadence (steps/min), and stride length (cm) of MS subjects using FeetMe^®^ and GAITRite^®^ devices. The exploratory objective was description of the correlation between gait variables (ambulation time, velocity, cadence, stride length) obtained using GAITRite^®^ devices and the clinical parameters (type of MS, EDSS score, number of relapses in the previous year, topography of current symptoms) including time of MS evolution (time since the first symptoms to inclusion in the study, in years) and annual relapse rate (ARR) of subjects with MS.

### Design and settings

This observational, cross-sectional, single center study was conducted between September 2018 and April 2019. The study recruited people diagnosed with MS consecutively at the Vithas Nisa Hospital in Seville, Spain. The people aged 18 to 55 years, diagnosed with MS according to McDonald 2010 criteria [22], with EDSS scores of 0–6.5, relapse free within 30 days at baseline, and agreed to wear footwear (in accordance with the specifications of FeetMe^®^) were consecutively recruited. People with a neurological disorder or any other concomitant disorder (apart from MS that affects walking) or using orthosis were excluded from the study. These study subjects were recruited at the study site, where the GAITRite^®^ system was already placed, as usual practice. Information was collected in a single visit, without the need for follow-up visits, unless in some cases it was necessary to perform the 25FWT during the following visit scheduled according to clinical practice. The demographic and clinical characteristics were collected from the medical records, namely age, gender, body mass index (BMI), type of MS, EDSS score, number of relapses in the previous year, topography of current symptoms etc.

**Fig 1:**
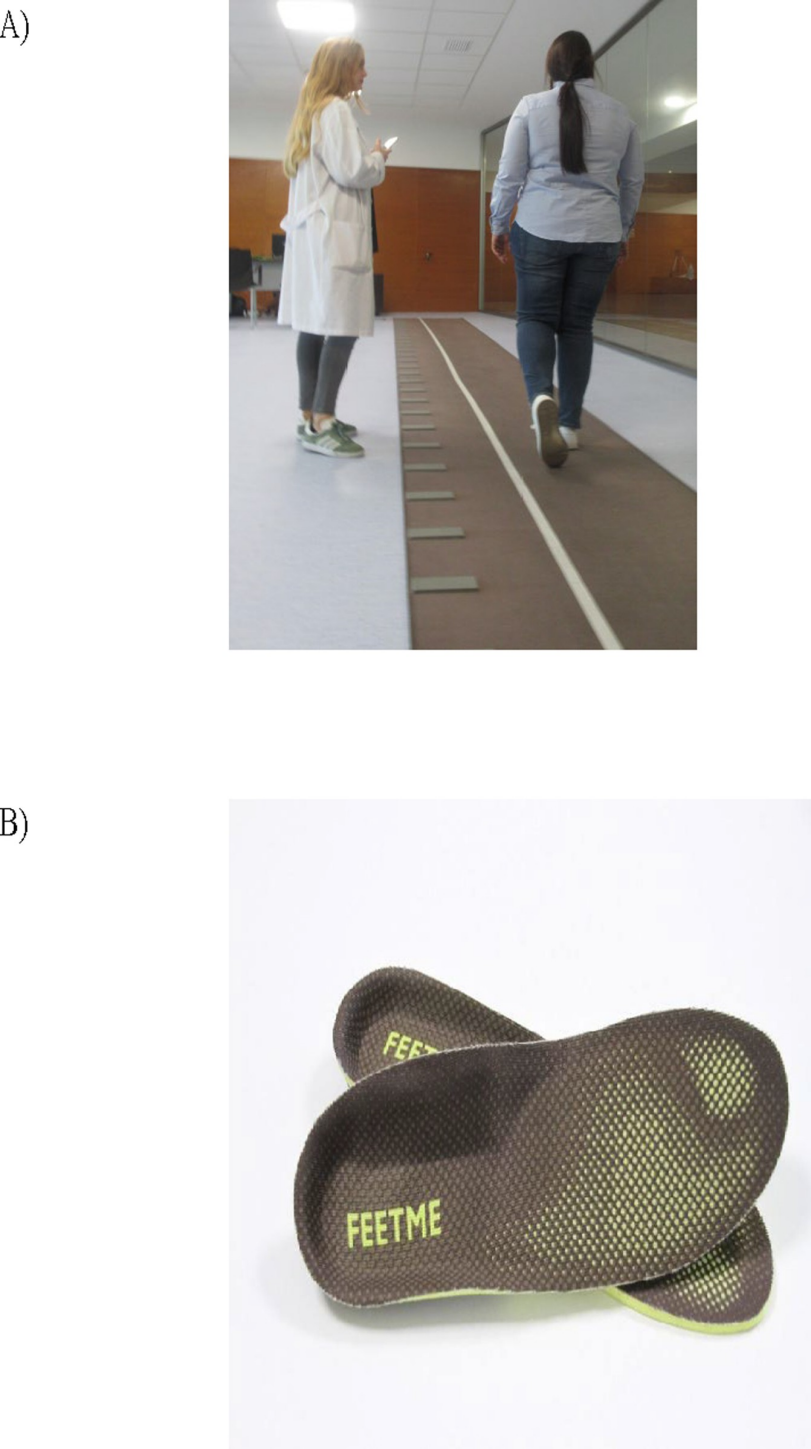
Devices to measure gait parameters (A) GAITRite^®^ and (B) FeetMe^®^ Monitor.

GAITRite^®^ is based on a gait corridor, implemented with sensors that record the pressure exerted in the footstep during gait. These are grouped in cells, which have an active area of 61 cm^2^ and contain 2,304 sensors arranged on a 48x48 grid (Fig 1A). FeetMe^®^ is an integrated sensor insole system that can be placed in any type of footwear used to measure gait spatiotemporal parameters and plantar pressure. This system combines 19 pressure sensors and inertial measurement unit (composed of an accelerometer and gyroscope). Subjects were wearing the FeetMe^®^ insoles while walking on the GAITRite^®^ mat. Both systems (GAITRite^®^ and FeetMe^®^) do not require anthropometric measures to calibrate measurements and version 2 of the FeetMe^®^ was used in the study (Fig 1B).

### Study endpoints

All endpoints by GAITRite^®^ device were obtained directly by variables recorded in the electronic case report form (eCRF). All endpoints by FeetMe^®^ device were calculated using the step-by-step variables file generated by FeetMe^®^ during the 25FWT (uploaded as part of the eCRF). The gait parameters evaluated are described in Table 1.

**Table 1:** Gait parameters recorded by GAITRite^®^ and FeetMe^®^ Monitor devices.

Variables (units)	Side	Definition	Decision for analysis	Range
**Primary Endpoint**				
Velocity 1 and 2 (cm/sec)	NA	It was obtained after dividing the distance traveled by the ambulation time. Velocity 1 was recorded directly from the device. Every patient had more than one velocity, therefore the velocity 2 is the mean for all recorded velocities.	NA	0–200
**Secondary Endpoints**				
**General Parameters**				
Ambulation time (sec)	NA	GAITRite^®^: It is the time elapsed between the heel strike of the first footprint and the heel strike of the last footprint. FeetMe^®^: It is time elapsed between the first heel strike of the first stride and the second heel strike of the last stride.	NA	0–200
Cadence 1 and 2 (steps/min)	NA	The number of steps within a minute. Cadence 1 was recorded directly from the device. Every patient had more than one cadence, therefore the cadence 2 is the mean for all recorded cadences.	NA	0–200
**Spatial Parameters**				
Stride length (cm)	Left	It is measured on the line of progression between the heel points of two consecutive footprints of the same foot (left to left, right to right).	As detailed below step length for FeetMe^®^ was calculated.** Stride length is a parameter complementing step length and it was obtained with GAITRite^®^ and FeetMe^®^ devices	
	Right			
	Total*			

*Total stride length = (Left stride + Right stride)/2; NA = Not applicable.

** Step length (cm) is measured along the line of progression from the heel center of the current footprint to the heel center of the previous footprint on the opposite foot.

Gait velocity for FeetMe^®^ was calculated using two formulas:

-
$\text{Velocity}\;1\left(\frac{\text{cm}}{\sec}\right)=\frac{\text{Distance}}{\text{Ambulation}\;\text{time}}$

-
$\text{Velocity}\;2\left(\frac{\text{cm}}{\sec}\right)=100*\text{Mean}(\text{Velocity}\;1)$

Ambulation time (sec) in the T25WT with both devices was presented according to GAITRite^®^ device and FeetMe^®^ calculations. Gait cadence for FeetMe^®^ was calculated using two formulas:

-
$\text{Cadence}\;1\left(\frac{\text{steps}}{\min}\right)=60*\frac{\text{Number}\;\text{of}\;\text{steps}}{\text{Ambulation}\;\text{time}}$

-
$\text{Cadence}\;2\left(\frac{\text{steps}}{\min}\right)=\text{Mean}\;(\text{Cadence}\;1)$

Stride length was analyzed for right and left joined values (total stride length which is calculated as [Left stride + Right stride]/2). This parameter was also presented as the average for left and right values for GAITRite^®^ and FeetMe^®^ devices.

### Study analysis sets

#### T25WT-both devices subject group (25SG)

The population consisted of the subjects included in the study who fulfill all the selection criteria and who had performed T25WT. In this group, subjects had performed T25WT when he/she had values recorded in the T25WT for GAITRite^®^ and FeetMe^®^ devices, including subjects who, for any reason, did not correctly perform or complete the test. Analysis of parameters included all data without excluding out-of-range data.

#### T25WT-both devices subject group with valid data (25SG+VD)

The evaluable population included subjects who meet all the selection criteria and who had performed T25WT with both devices and had valid and evaluable data, at least for velocity parameter. The selection criteria were:

- Meeting all eligibility criteria
- T25WT correctly performed (a “yes” in question regarding a successful test)
- Difference between date of visit and date of T25WT < 30 days, in case that T25WT is performed in a different day Available and valid data for both devices (at least for velocity) including number of total steps recorded in each device were the same and data for velocity registered in each device was on-plausible-range (GAITRite^®^ and FeetMe^®^), excluding out-of-range data

#### T25WT intended subject group (25ISG)

This population consisted of all the subjects included in the study, who were intended to perform the T25WT.

### Statistical analysis

The continuous variables were summarized with N, mean, standard deviation (SD), minimum, 25^th^ percentile (1^st^ quartile), median, 75^th^ percentile (3^rd^ quartile), and maximum, while categorical variables were summarized with frequency and percentage of subjects per response category. Intra-class correlation coefficient (ICC) was used to assess the test-retest reliability [23]. To quantify the test-retest reliability, the closer the ICC is to 1.0, the higher is the reliability and the lower is the error variance. A ratio of 0.3–0.4 indicates fair agreement, 0.5–0.6 moderate agreement, 0.7–0.8 strong agreement, and >0.8 almost perfect agreement [24]. Also, Bland-Altman plot analysis for gait parameters was used to compare the two devices.

Evaluable subjects for each population (25SG and 25SG+VD) were described in terms of socio-demographic and clinical characteristics. Univariate and multivariate general linear model (GLM) regression models were constructed to assess the impact of subject characteristics on gait parameters. Multivariable regression models were constructed for each gait parameter (obtained by GAITRite^®^), adjusting for those independent variables (such as age, gender, BMI, type of MS, time to first symptoms, symptomatology, and treatments for MS) associated with the dependent variable with a *p* value<0.1 at univariate level. Variables significant at an alpha of 0.05 were retained as predictors, using a stepwise approach for both populations.

### Sensitivity analysis

Sensitivity analysis was performed to obtain level of agreement between GAITRite^®^ and FeetMe^®^ devices taking into account different sub-populations i.e.:

- Excluding outliers for both the devices in 25SG and 25SG+VD population,
- Excluding out-of-range values for both the devices in 25SG and 25SG+VD population,
- Excluding uncertain values (stride length) and all uncertain values in FeetMe^®^ device for 25SG population in post-hoc analysis (excluding 2 meters steps).

Out-of-range values for the gait parameters were considered according to the criteria specified in Table 1. Using the definition of outlier as an observation that lies outside the overall pattern of a distribution, outliers were considered any value which fell more than 1.5 times the interquartile range above the third quartile or below the first quartile [25]. Reporting analysis on time parameters, asymmetry parameters, and H-H base of support or base width (cm) is beyond the scope of the article.

### Ethical consideration

Protocol and study site received the approval from the Institutional Review Board of the Hospital Virgen de la Macarena (Sevilla), Spain and written informed consent was obtained from each participating subject prior to proceeding with the study. The clinicians and other research staff involved in the study complied with the Declaration of Helsinki following the local regulations (including privacy laws).

## Results

### Baseline characteristics of subjects

**Fig 2:**
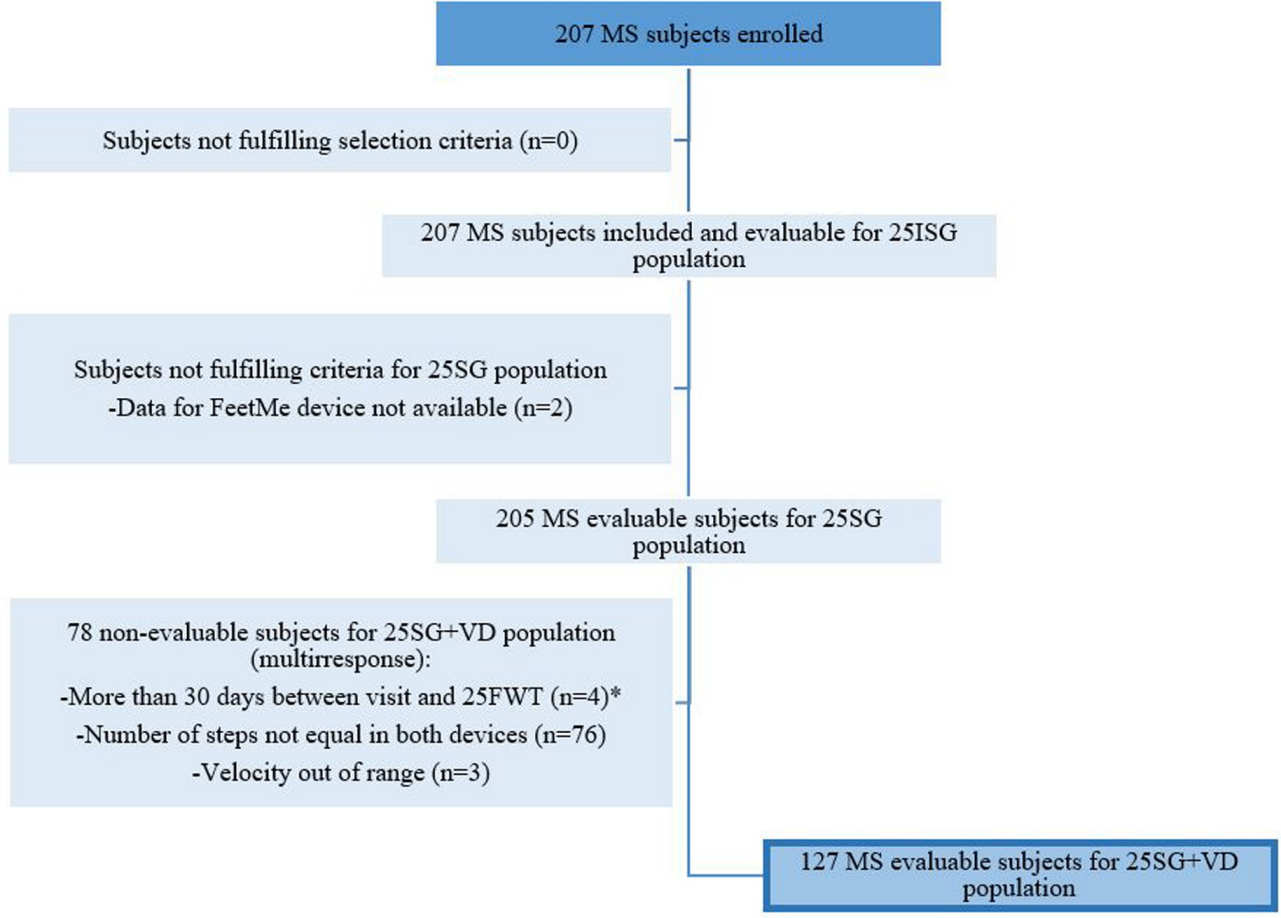
Flowchart of subject disposition. 25SG = T25WT-both devices subject group; 25SG+VD = T25WT-both devices subject group with valid data; 25ISG = T25WT intended subject group; MS = Multiple sclerosis; T25WT = times 25-feet walk. *10 subjects did not perform 25FWT the same day due to following reasons: Malfunction or alteration of the features / benefits of FeetMe^®^, Malfunction or alteration of the characteristics / benefits of GAITRite, Reasons intrinsic to the subject (inability to walk, does not finish the 8 m of the test, etc.), Problems unrelated to the device and the subject (operation of the service, etc.), Withdrawal of informed consent by the subject, other reasons.

The number of subjects in each analysis population (i.e., 25ISG, 25SG, and 25SG+VD) is summarized in Fig 2. A total of 207 study subjects with MS were enrolled in the study and included in the 25ISG. Of them, 205 were considered in 25SG group and 127 subjects in 25SG+VD group. At baseline, mean (SD) age of subjects with MS in the 25SG population was 41.5 (8.0) years and 137 (66.8%) were females (Table 2). The mean (SD) BMI was 24.7 (4.5) Kg/m^2^, and majority of the study subjects had relapsing-remitting MS (RRMS) (n = 170; 82.9%). Mean (SD) time since MS diagnosis was 8.1 (7.0) years and MS evolution since first symptoms was 11.7 (8.5) years. Mean (SD) EDSS score was 3.1 (2.0) and 96 (46.8%) subjects had an EDSS from 4.0 to 6.5, and 32 (15.6%) subjects used walking stick or crutch for support to perform the T25WT.

**Table 2:** Demographic and clinical characteristics of the different study population.

Variables	Category	25ISG	25SG	25SG+VD
**Socio-demographic characteristic**				
**Gender**	Total, n	207	205	127
	Male, n (%)	68 (32.9)	68 (33.2)	41 (32.3)
	Female, n (%)	139 (67.1)	137 (66.8)	86 (67.7)
**Age (years)**	Mean (SD)	41.55 (8.0)	41.47 (8.0)	40.66 (8.2)
**BMI (Kg/m** ^**2**^ **)**	Mean (SD)	24.7 (4.5)	24.7 (4.5)	24.9 (4.6)
**Leg length Left (cm)**	Mean (SD)	86.2 (6.0)	86.2 (6.0)	85.7 (6.1)
**Leg length Right (cm)**	Mean (SD)	86.1 (6.1)	86.1 (6.0)	85.7 (6.2)
**Leg length (cm)**	Mean (SD)	86.1 (6.0)	86.1 (6.0)	85.7 (6.1)
**Clinical characteristics**				
**Type of MS**	RRMS, n (%)	170 (82.1)	170 (82.9)	106 (83.5)
	PPMS, n (%)	18 (8.7)	17 (8.3)	11 (8.7)
	SPMS, n (%)	19 (9.2)	18 (8.8)	10 (7.9)
**Time since first symptoms (years)**	Mean (SD)	11.70 (8.5)	11.71 (8.5)	11.26 (8.5)
**Time since MS diagnosis (years)**	Mean (SD)	8.11 (7.0)	8.09 (7.0)	7.90 (7.0)
**EDSS**	0, n (%)	14 (6.8)	14 (6.8)	11 (8.7)
	1.0, n (%)	31 (15.0)	31 (15.1)	22 (17.3)
	1.5, n (%)	33 (15.9)	33 (16.1)	22 (17.3)
	2.0, n (%)	16 (7.7)	16 (7.8)	14 (11.0)
	2.5, n (%)	5 (2.4)	5 (2.4)	3 (2.4)
	3.0, n (%)	5 (2.4)	5 (2.4)	2 (1.6)
	3.5, n (%)	5 (2.4)	5 (2.4)	3 (2.4)
	4.0, n (%)	36 (17.4)	36 (17.6)	23 (18.1)
	4.5, n (%)	11 (5.3)	11 (5.4)	8 (6.3)
	5.0, n (%)	7 (3.4)	7 (3.4)	2 (1.6)
	5.5, n (%)	10 (4.8)	10 (4.9)	4 (3.1)
	6.0, n (%)	12 (5.8)	12 (5.9)	4 (3.1)
	6.5, n (%)	22 (10.6)	20 (9.8)	9 (7.1)
**EDSS**	Median (Q_1_; Q_3_)	3.0 (1.5; 4.5)	3.0 (1.5; 4.5)	2.0 (1.0; 4.0)
	Min; Max	(0.0; 6.5)	(0.0; 6.5)	(0.0; 6.5)
**Relapses in the last year**	Without relapses, n (%)	152 (73.4)	150 (73.2)	92 (72.4)
	With relapses, n (%)	51 (24.6)	51 (24.9)	32 (25.2)
	1-relapse, n (%)	35 (17.2)	35 (17.4)	23 (18.5)
	2-relapses, n (%)	13 (6.4)	13 (6.5)	7 (5.6)
	3-relapses, n (%)	3 (1.5)	3 (1.5)	2 (1.6)
	NA, n (%)	4 (1.9)	4 (1.9)	3 (2.3)
Number of relapses in the last year*	Mean (SD)	0.3 (0.7)	0.4 (0.7)	0.4 (0.7)
Time since last relapse (years)*	Mean (SD)	0.5 (0.3)	0.5 (0.3)	0.4 (0.3)
**Presence of current MS symptoms**	No, n (%)	12 (5.8)	11 (5.4)	9 (7.1)
	Yes, n (%)	195 (94.2)	194 (94.6)	118 (92.9)
**Current symptoms**	Sensitive, n (%)	92 (44.4)	92 (44.9)	55 (43.3)
	Visuals, n (%)	21 (10.1)	21 (10.2)	17 (13.4)
	Motors, n (%)	107 (51.7)	106 (51.7)	57 (44.9)
	Brain stem, n (%)	52 (25.1)	51 (24.9)	29 (22.8)
	Sphincter alteration, n (%)	27 (13.0)	27 (13.2)	16 (12.6)
	Cognitive disorders, n (%)	2 (1.0)	2 (1.0)	1 (0.8)
**Type of support to perform T25WT**	None, n (%)	175 (84.5)	173 (84.4)	115 (90.6)
	Walking stick, n (%)	11 (5.3)	11 (5.4)	5 (3.9)
	Crutch, n (%)	21 (10.1)	21 (10.2)	7 (5.5)

* This information is presented only for those who had had a relapse in the past year. 25SG = T25WT-both devices subject group; 25SG+VD = T25WT-both devices subject group with valid data; 25ISG = T25WT intended subject group; BMI = Body mass index; cat = category; EDSS = Expanded Disability Status Scale; MS = multiple sclerosis; NA = Data not available; PPMS = Primary progressive MS; RRMS = Relapsing-Remitting MS; SPMS = Secondary-progressive MS; Q_1 =_ 1st quartile; Q_3_ = 3rd quartile; SD = Standard deviation; T25WT = Timed 25-Foot Walk.

### Description and correlation of gait parameters

In one subject, velocity (calculated using formula 1) could not be obtained because values of distance/ambulation time were not correctly recorded. In 25SG population, mean (SD) velocity 1 was 98.9 cm/sec (35.9) for GAITRite^®^ and 103.3 cm/sec (32.3) for FeetMe^®^, with a strong ICC (0.833) between the two devices. Similarly, the mean (SD) velocity 2 was 98.6 cm/sec (36.1) and 104.1 cm/sec (33.2) for GAITRite^®^ and FeetMe^®^, respectively with 0.865 ICC. Mean (SD) ambulation time was relatively longer with GAITRite^®^ (9.7 sec [8.2]) compared to FeetMe^®^ (9.6 sec [7.5]). Whereas mean stride length (SD) was shorter with GAITRite^®^ (119.0 cm [26.4]) compared to FeetMe^®^ (126.0 cm [42.1]). Velocity (1 and 2), ambulation time and cadence (1 and 2), showed a statistically significant and very strong agreement between gait parameters obtained for FeetMe^®^ and GAITRite^®^ (ICC > 0.800). All estimated gait parameters for 25SG and 25SG+VD population are summarized in Table 3.

**Table 3:** Statistics obtained in the paired analysis (25SG and 25SF+VD).

Parameter	N	GAITRite^®^ mean (SD)	FeetMe^®^ Monitor mean (SD)	Difference mean (SD)	ICC	[95% IC]
**25SG population**						
Velocity 1 (cm/sec)	204	98.9 (35.9)	103.3 (32.3)	-4.4 (19.4)	0.833	[0.147–1.520]
Velocity 2 (cm/sec)	205	98.6 (36.1)	104.1 (33.2)	-5.5 (17.3)	0.865	[0.179–1.550]
Ambulation time (sec)	205	9.7 (8.2)	9.6 (7.5)	0.1 (2.9)	0.932	[0.246–1.620]
Cadence 1 (steps/min)	205	96.7 (21.9)	96.7 (21.9)	0.04 (9.4)	0.908	[0.222–1.590]
Cadence 2 (steps/min)	205	96.7 (21.9)	99.5 (21.5)	-2.8 (9.3)	0.901	[0.215–1.590]
Stride length Left (cm)	205	118.7 (26.4)	120.8 (29.4)	-2.1 (22.6)	0.673	[-0.013–1.360]
Stride length Right (cm)	204	119.0 (26.5)	130.9 (75.5)	-11.9 (76.9)	0.068	[-0.618–0.754]
Stride length (cm)	204	119.0 (26.4)	126.0 (42.1)	-7.0 (41.2)	0.301	[-0.385–0.987]
**25SG+VD population**						
Velocity 1 (cm/sec)	127	104.5 (31.6)	107.6 (30.3)	-3.2 (14.7)	0.883	[0.197–1.570]
Velocity 2 (cm/sec)	127	104.5 (31.6)	108.9 (31.0)	-4.5 (13.4)	0.899	[0.213–1.580]
Ambulation time (sec)	127	8.07 (5.0)	8.07 (5.1)	-0.0 (0.3)	0.998	[0.312–1.680]
Cadence 1 (steps/min)	127	100.4 (17.2)	100.7 (17.5)	-0.3 (2.7)	0.988	[0.302–1.670]
Cadence 2 (steps/min)	127	100.4 (17.2)	102.9 (17.9)	-2.5 (2.7)	0.978	[0.292–1.660]
Stride length Left (cm)	127	122.6 (23.9)	122.8 (22.1)	-0.3 (9.4)	0.918	[0.232–1.600]
Stride length Right (cm)	127	122.7 (24.1)	127.7 (37.9)	-5.0 (36.3)	0.343	[-0.343–1.030]
Stride length (cm)	127	122.6 (24.0)	125.3 (25.5)	-2.6 (19.8)	0.679	[-0.007–1.370]

25SG = T25WT-both devices subject group; 25SG+VD = T25WT-both devices subject group with valid data; ICC = Intra-class correlations; SD = Standard deviation; Criteria for agreement (ICC): 0<ICC<0.3 = Poor; 0.3≤ICC<0.5 = Fair; 0.5≤ICC<0.7 = Moderate; 0.7≤ICC<0.8 = Strong; ICC≥0.8 = Almost perfect.

### Scatter and Bland & Altman plots for gait parameters obtained by GaitRite^®^ and FeetMe^®^

**Fig 3:**
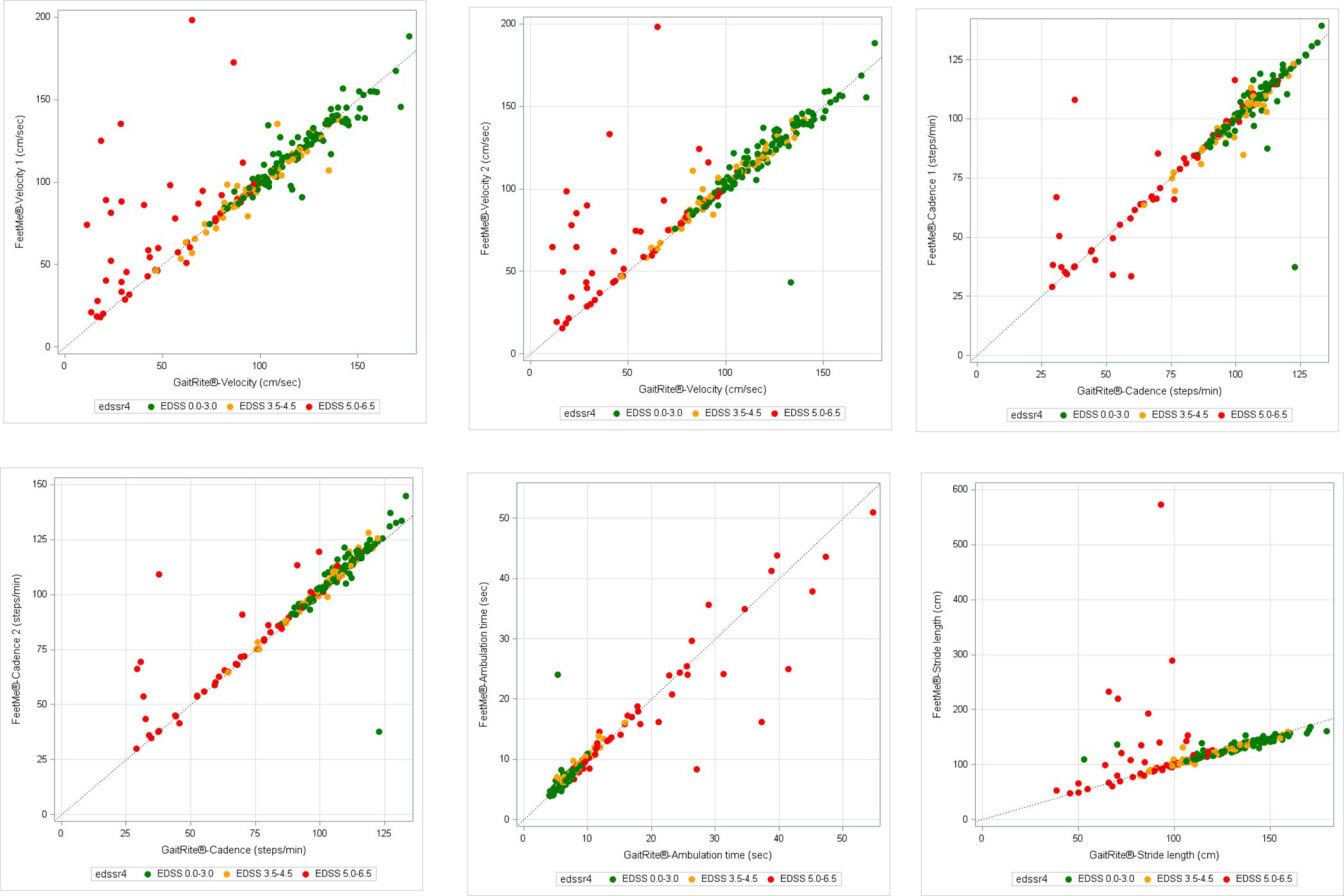
Scatter plots for gait parameters obtained by GAITRite^®^ and FeetMe^®^ Monitor devices in 25SG population. (A) Velocity 1 (cm/sec) (B) Velocity 2 cm/sec (C) Cadence 1 (steps/min) (D) Cadence 2 (steps/min) (E) Ambulation time (sec) (F) Stride length (cm). EDSS = Expanded Disability Status Scale; x = y is the line of equality between GAITRite^®^ and FeetMe^®^ Monitor.

ICC were also analyzed by EDSS subgroups 25SG population (Fig 3). Velocity, cadence, and stride length showed that subjects with low disability grade (EDSS 0–3.0) have higher values detected by GAITRite^®^ and showed a very good fit with FeetMe^®^ (Fig 3A–3C and 3E). Conversely, ambulation time analysis showed that subjects with low disability grade had lower values detected by GAITRite^®^ but also showed a very good fit with FeetMe^®^ (Fig 3F). In addition, high disability (EDSS 5–6.5) was associated with lower agreement between GAITRite^®^ and FeetMe^®^.

The Bland and Altman plots for 25SG population and percentage of values out of limits of agreement are shown in S1 Fig. There was a much greater deviation in stride length differential: it was not observed so much in the individual steps (right and left), but a greater difference was obtained for difference between Left/Right (S1 Fig).

### Sensitivity analysis outcomes

The level of agreement between GAITRite^®^ and FeetMe^®^ for all the endpoints while considering different sub-populations is shown in Supporting information (S1 Table). The ICC (≥0.8) obtained for velocity, ambulation time and cadence (1 and 2) indicates an almost perfect agreement between both the devices.

### Relation of gait parameters with characteristics of study subjects

GAITRite^®^ was used to define gait parameters, as the gold standard. There was no strong association between time of MS evolution and gait parameters. A strong association was observed between EDSS score and velocity, cadence and stride length. Velocity, cadence, step, and stride length decreased for high EDSS score (5.0–6.5). However, no association was observed between the number of relapses (from 0 to 3) in the last year and GAITRite^®^ parameters. The diagnosis of secondary-progressive MS (SPMS) was associated with decreased velocity (decreased by 15.5 cm/sec) and increased ambulation time (increased by 6.5 sec) in 25SG population. While, in 25SG+VD population, the diagnosis of SPMS presented a significant association with ambulation time (increases 3.5 sec) and cadence (decreases 9.9 steps/min). In multivariate model, velocity measured by GAITRite^®^ was depended up on the following factors: EDSS 5–6.5 (decreased velocity by 59 cm/sec), SPMS (decreased velocity by 15 cm/sec), motor symptoms (decreased velocity by 10.7 cm/sec) and to be female (decreased velocity by 8.5 cm/sec). In 25SG+VD, subjects with high EDSS (5–6.5) had lower velocity, cadence, and stride length. A summary of significant values (p<0.05) obtained in multivariate models for other parameters in 25SG and 25SG+VD population is shown in Table 4.

**Table 4:** Multivariate models for each gait parameters by GAITRite^®^ device (25SG and 25SG+VD).

	Female gender (p value)	EDSS 2.5–4.5 (p value)	EDSS 5.0–6.5 (p value)	SPMS (p value)	Motor symptoms (p value)	Brain stem (p value)	Cognitive disorders (p value)
Reference group	Male gender	EDSS 0.0–2.0	EDSS 0.0–2.0	RRMS	-	-	-
**25SG population**							
**Velocity (cm/sec)**	-8.5 (0.01)	-	-59.1 (<0.01)	-15.5 (0.01)	-10.7 (0.002)	-	-
**Ambulation time (sec)**	-	-	11.3 (<0.01)	6.5 (<0.01)	-	-2.1 (0.03)	9.8 (0.02)
**Cadence (steps/min)**	-	-	-31.1 (<0.01)	-	-4.6 (0.01)	-	-
**Stride length (cm)**	-14.1 (0.01)	-12.6 (0.003)	-42.0 (<0.01)	-	-6.0 (0.02)	-	-
**25SG+VD population**							
**Velocity (cm/sec)**	-8.5 (0.04)	-17.9 (<0.01)	-62.0 (<0.01)	-	-9.1 (0.02)	-	-
**Ambulation time (sec)**	-	1.7 (0.01)	8.7 (<0.01)	3.5 (0.001)	-	24.0 (<0.01)	-
**Cadence (steps/min)**	-	-7.8 (0.004)	-35.6 (<0.01)	-9.9 (0.03)	-	-	-
**Stride length (cm)**	-14.9 (<0.01)	-14.1 (<0.01)	-37.5 (<0.01)	-	-	-50.0 (0.02)	-

25SG = T25WT-both devices subject group; 25SG+VD = T25WT-both devices subject group with valid data; EDSS = Expanded Disability Status Scale; RRMS = Relapsing-remitting multiple sclerosis; SPMS = Secondary-progressive multiple sclerosis.

## Discussion

In our study, a shoe insole device (FeetMe^®^ Monitor) with integrated sensors was used to perform a comprehensive and objective assessment of gait alterations. Simultaneously, this insole device was also compared with the results obtained by the reference system (GAITRite^®^) to validate its use in patients with MS. The assessment of gait in patients with MS provides an understanding of the change in gait parameters over the course of disease, defining gait patterns for different stages of the disease and, therefore, detect objective signs of worsening or progression beforehand.

In our study, FeetMe^®^ Monitor assessed variables which are of clinical relevance and used in measurement of gait in MS patients. Gait disorders in MS patients are commonly measured as maximum free walking distance in the EDSS or as decline in maximum walking speed in timed walks by T25WT [9]. FeetMe^®^ Monitor obtained a high degree of precision and agreement with the data obtained by GAITRite^®^. The study also suggested the velocity at which the subjects performed the T25WT was very similar between both devices used, showing an almost perfect agreement. Gait velocity is a valid, sensitive, and reliable measure used widely in both clinical and research settings. It assesses functional capacity and predicts functional decline in various health conditions [26–28]. The research findings continue to validate the prognostic and predictive utility of gait velocity, and which is also referred to as the “sixth vital sign” [29]. Gait velocity is appropriate to monitor functional status and the current study used this parameter as the primary endpoint to determine the concordance and statistical precision between GAITRite^®^ and FeetMe^®^ devices in the study subjects with a broad range of disability status (0–6.5 EDSS). Similar correlations were observed in a study by Howell et al, where data simultaneously collected from clinical motion analysis laboratory and insole sensor for ground reaction force and ankle moment were highly correlated (ICC >0.95) [30].

Good agreement (over 0.9) between FeetMe^®^ Monitor and GAITRite^®^ were also reported for other parameters that neurologists consider important in monitoring the gait of MS patients, such as ambulation time and cadence.

In EDSS subgroup analysis, gait parameters such as velocity, cadence and stride length showed lower agreement for the group with the highest EDSS. This low agreement can be accredited to the fact that either FeetMe^®^ or GAITRite^®^ are less reliable with more disabled people than with less disabled people.

The sensitivity analysis showed that excluding outliers and out-of-range values of each gait parameter for GAITRite^®^ and FeetMe^®^ Monitor, agreement between the devices was even better for velocity (according to definition 1 and 2), ambulation time, and cadence (according to definition 1 and 2). Similarly, stride length also showed strong agreement between the devices, with high ICC.

The exploratory analysis of the relationship between gait characteristics and disease severity suggested a strong association between EDSS scores and velocity, cadence, stride and step length. For study subjects with high EDSS score (5.0–6.5), velocity, cadence, step and stride length were lower. However, no association was observed between number of relapses (from 0 to 3) in the last year and GAITRite^®^ parameters.

The EDSS scale is a non-quantitative ordinal scale. Therefore, additional quantitative measurements are important in assessing disease symptoms such as gait. Both the GAITRite^®^ and FeetMe^®^ Monitor systems were able to provide accurate measurement of gait which cannot be obtained by only using clinical scale such as EDSS. Comparing the utility of both systems, FeetMe^®^ Monitor, allows greater flexibility of use than GAITRite^®^ without losing the accuracy and reliability of the assessment. FeetMe^®^ Monitor is associated with characteristics that are usually shown by an effective laboratory gait device such as seamless use, easy transport, and quick installation. Although current study has been carried out under laboratory settings, the FeetMe^®^ Monitor technology can be applied under home setting conditions. Saito et al have also highlighted the advantages of using pressure-sensitive foot insoles for gait analysis in terms of inherent ease of wearability and portability (in comparison to traditional instrumentations such as pedobarographs and force platforms) [31].

The study also emphasized on the role of foot insoles in making gait recordings between the scenarios of activities of daily living [31]. Other studies that have used pressure sensor technology to evaluate gait impairment in patients with MS suggested that insole pressure sensors are sensitive enough to capture gait dysfunction in patients with minimal or no disability [32–34]. Similar to pressure sensors, inertial sensors are the widely used type of wearable devices for gait and balance analysis and have been validated and have shown some limitations in patients with walking impairments in the ability to segment steps [35,36]. According to a critical review by Shanahan et al, the wearable insole sensors allow gait measurement in a patient’s regular surroundings for extended periods of time and send gait data remotely to the laboratory or clinic. The connectivity with the smartphones and watches further improves the usability of this device among patients and treating clinicians [19].

The limitations of the study include difficulty in matching of the gait readings by both the devices because the measurements did not start and end at the exact same time. This difficulty was minimized by imparting training to the researchers responsible for the subjects’ evaluation, because after the data collection, synchronization was not possible and GAITRite^®^ parameters could not be recalculated. It was not possible to perform step-by-step analysis (as metrics were available for FeetMe^®^ Monitor but not for GAITRite). In the present study, data was obtained from single center, that regularly uses GAITRite^®^. If there were differences across geographically diverse regions with variable clinical practice patterns, this could introduce a high degree of variability into the data. However, this should not be a limitation to extrapolate the results given that the test is performed during a routine visit consisting in walking on GAITRite^®^ and collecting data by means of software for both devices, in an objective way.

## Conclusions

FeetMe^®^ Monitor is an insole sensor device used in comprehensive and objective assessment of gait impairment. It provides quantitative measures with greater flexibility than GAITRite^®^ in both clinic and research settings. This validation study was carried out to authenticate the FeetMe^®^ Monitor in routine clinical practice for a heterogeneous population of MS patients in Spain with walking impairments. According to the study, agreement between the GAITRite^®^ (classic reference system) and FeetMe^®^ Monitor was “almost perfect” in velocity, ambulation, cadence, and stride length parameters. The consistency in the value of gait parameters and review of existing scientific literature demonstrate that FeetMe^®^ Monitor is a valid and reliable device for gait analysis in MS patients. It is the first validated medical device that would allow a portable monitoring of the gait of MS patients. In addition, FeetMe^®^ Monitor device is a transportable and field-usable alternative for the assessment of the characteristics of gait in the neurologist’s visits. The use of the device is safe, fairly simple and yet technically elaborated. Due to these qualities, it can lead to improved patients’ engagement in assessment and rehabilitation and could result in reduced clinic visits by providing real-time information. Further studies are needed to better understand the usability of FeetMe^®^ Monitor in gait monitoring during the course of disease and to support the diagnosis of acute exacerbations (relapses), fluctuations (paroxysmal symptoms), and possible evaluation of responses to symptomatic treatments and disease modifying therapies. For future studies, the identification of transition markers of disease progression would be important. In fact, further knowledge of gait disorders will allow for physiotherapeutic corrections and an assessment of therapies that can modify those previously detected alterations. In addition, the application of FeetMe^®^ Monitor could potentially be extended to other research studies as an objective measure of gait characterization and an inference of disease evolution.

## Supporting information

S1 Fig Bland-Altman plots for 25SG population. LOA = Limits of Agreement.
(TIFF)

S1 Table Agreement between different gait parameters for GAITRite^®^ and FeetMe^®^ Monitor devices within sub-populations. 25SG = T25WT-both devices subject group; 25SG+VD = T25WT-both devices subject group with valid data; ICC = Intra-class correlations; SD = Standard deviation; Criteria for agreement (ICC): 0<ICC<0.3 = Poor; 0.3≤ICC<0.5 = Fair; 0.5≤ICC<0.7 = Moderate; 0.7≤ICC<0.8 = Strong; ICC≥0.8 = Almost perfect.
(DOCX)

## References

[1] Cameron MH, Wagner JM. Gait abnormalities in multiple sclerosis: pathogenesis, evaluation, and advances in treatment. Current neurology and neuroscience reports. 2011;11(5):507–15. Epub 2011/07/23. doi: 10.1007/s11910-011-0214-y . 21779953

[2] GBD 2016 Multiple Sclerosis Collaborators. Global, regional, and national burden of multiple sclerosis 1990–2016: a systematic analysis for the Global Burden of Disease Study 2016. The Lancet Neurology. 2019;18(3):269–85. Epub 2019/01/27. doi: 10.1016/S1474-4422(18)30443-5 ; PubMed Central PMCID: PMC6372756. 30679040 PMC6372756

[3] Bártulos Iglesias M, Marzo Sola ME, Estrella Ruiz LA, Bravo Anguiano Y. Epidemiological study of multiple sclerosis in La Rioja. Neurologia. 2015;30(9):552–60. Epub 2014/07/01. doi: 10.1016/j.nrl.2014.04.016 . 24975346

[4] Moreno-Torres I, Sabín-Muñoz J, García-Merino A. Chapter 1—Multiple Sclerosis: Epidemiology, Genetics, Symptoms, and Unmet Needs Available from: https://pubs.rsc.org/en/content/chapterhtml/2019/bk9781788014502-00001?isbn=978-1-78801-450-2.2019.

[5] Paltamaa J, Sarasoja T, Leskinen E, Wikström J, Mälkiä E. Measures of physical functioning predict self-reported performance in self-care, mobility, and domestic life in ambulatory persons with multiple sclerosis. Archives of physical medicine and rehabilitation. 2007;88(12):1649–57. Epub 2007/12/01. doi: 10.1016/j.apmr.2007.07.032 . 18047881

[6] Pirker W, Katzenschlager R. Gait disorders in adults and the elderly: A clinical guide. Wiener klinische Wochenschrift. 2017;129(3–4):81–95. Epub 2016/10/23. doi: 10.1007/s00508-016-1096-4 ; PubMed Central PMCID: PMC5318488. 27770207 PMC5318488

[7] Kurtzke JF. Rating neurologic impairment in multiple sclerosis: an expanded disability status scale (EDSS). Neurology. 1983;33(11):1444–52. Epub 1983/11/01. doi: 10.1212/wnl.33.11.1444 . 6685237

[8] Noseworthy JH. Clinical scoring methods for multiple sclerosis. Annals of neurology. 1994;36 Suppl:S80–5. Epub 1994/01/01. doi: 10.1002/ana.410360718 . 8017893

[9] Bethoux F, Bennett S. Evaluating walking in patients with multiple sclerosis: which assessment tools are useful in clinical practice? International journal of MS care. 2011;13(1):4–14. Epub 2011/04/01. doi: 10.7224/1537-2073-13.1.4 ; PubMed Central PMCID: PMC3882949. 24453700 PMC3882949

[10] Bin Sawad A, Seoane-Vazquez E, Rodriguez-Monguio R, Turkistani F. Evaluation of the Expanded Disability Status Scale and the Multiple Sclerosis Functional Composite as clinical endpoints in multiple sclerosis clinical trials: quantitative meta-analyses. Curr Med Res Opin. 2016;32(12):1969–74. Epub 2016/09/08. doi: 10.1080/03007995.2016.1222516 . 27603119

[11] Luzzio C, Dangond F. What are the advantages and limitations of the 10-point Kurtzke Expanded Disability Status Scale (EDSS) in multiple sclerosis (MS)? 2019. Available from: https://www.medscape.com/answers/1146199-5753/what-are-the-advantages-and-limitations-of-the-10-point-kurtzke-expanded-disability-status-scale-edss-in-multiple-sclerosis-ms.

[12] Bethoux FA, Palfy DM, Plow MA. Correlates of the timed 25 foot walk in a multiple sclerosis outpatient rehabilitation clinic. International journal of rehabilitation research Internationale Zeitschrift fur Rehabilitationsforschung Revue internationale de recherches de readaptation. 2016;39(2):134–9. Epub 2016/03/02. doi: 10.1097/MRR.0000000000000157 ; PubMed Central PMCID: PMC4850097. 26926380 PMC4850097

[13] Stellmann JP, Neuhaus A, Götze N, Briken S, Lederer C, Schimpl M, et al. Ecological validity of walking capacity tests in multiple sclerosis. PloS one. 2015;10(4):e0123822. Epub 2015/04/17. doi: 10.1371/journal.pone.0123822 ; PubMed Central PMCID: PMC4399985. 25879750 PMC4399985

[14] Abbadessa G, Lavorgna L, Miele G, Mignone A, Signoriello E, Lus G, et al. Assessment of Multiple Sclerosis Disability Progression Using a Wearable Biosensor: A Pilot Study. J Clin Med. 2021;10(6):1160. doi: 10.3390/jcm10061160 . 33802029 PMC8001885

[15] Lavorgna L, Iaffaldano P, Abbadessa G, Lanzillo R, Esposito S, Ippolito D, et al. Disability assessment using Google Maps. Neurol Sci. 2022;43(2):1007–14. Epub 2021/06/17. doi: 10.1007/s10072-021-05389-7 . 34142263 PMC8211455

[16] Jagos H, Pils K, Haller M, Wassermann C, Chhatwal C, Rafolt D, et al. Mobile gait analysis via eSHOEs instrumented shoe insoles: a pilot study for validation against the gold standard GAITRite(®). J Med Eng Technol. 2017;41(5):375–86. Epub 2017/06/03. doi: 10.1080/03091902.2017.1320434 . 28573909

[17] Steinert A, Sattler I, Otte K, Röhling H, Mansow-Model S, Müller-Werdan U. Using New Camera-Based Technologies for Gait Analysis in Older Adults in Comparison to the Established GAITRite System. Sensors (Basel, Switzerland). 2019;20(1). Epub 2019/12/28. doi: 10.3390/s20010125 ; PubMed Central PMCID: PMC6983253. 31878177 PMC6983253

[18] Webster KE, Wittwer JE, Feller JA. Validity of the GAITRite walkway system for the measurement of averaged and individual step parameters of gait. Gait Posture. 2005;22(4):317–21. Epub 2005/11/09. doi: 10.1016/j.gaitpost.2004.10.005 . 16274913

[19] Shanahan CJ, Boonstra FMC, Cofré Lizama LE, Strik M, Moffat BA, Khan F, et al. Technologies for Advanced Gait and Balance Assessments in People with Multiple Sclerosis. Frontiers in neurology. 2017;8:708. Epub 2018/02/17. doi: 10.3389/fneur.2017.00708 ; PubMed Central PMCID: PMC5799707. 29449825 PMC5799707

[20] Muro-de-la-Herran A, Garcia-Zapirain B, Mendez-Zorrilla A. Gait analysis methods: an overview of wearable and non-wearable systems, highlighting clinical applications. Sensors (Basel, Switzerland). 2014;14(2):3362–94. Epub 2014/02/22. doi: 10.3390/s140203362 ; PubMed Central PMCID: PMC3958266. 24556672 PMC3958266

[21] Farid L, Jacobs D, Do Santos J, Simon O, Gracies JM, Hutin E. FeetMe® Monitor-connected insoles are a valid and reliable alternative for the evaluation of gait speed after stroke. Topics in stroke rehabilitation. 2021;28(2):127–34. Epub 2020/07/14. doi: 10.1080/10749357.2020.1792717 . 32654627

[22] Polman CH, Reingold SC, Banwell B, Clanet M, Cohen JA, Filippi M, et al. Diagnostic criteria for multiple sclerosis: 2010 revisions to the McDonald criteria. Annals of neurology. 2011;69(2):292–302. Epub 2011/03/10. doi: 10.1002/ana.22366 ; PubMed Central PMCID: PMC3084507. 21387374 PMC3084507

[23] Liljequist D, Elfving B, Skavberg Roaldsen K. Intraclass correlation—A discussion and demonstration of basic features. PloS one. 2019;14(7):e0219854. Epub 2019/07/23. doi: 10.1371/journal.pone.0219854 ; PubMed Central PMCID: PMC6645485. 31329615 PMC6645485

[24] Weir JP. Quantifying test-retest reliability using the intraclass correlation coefficient and the SEM. J Strength Cond Res. 2005;19(1):231–40. Epub 2005/02/12. doi: 10.1519/15184.1 . 15705040

[25] Moore DS, McCabe GP. Introduction to the practice of statistics. 3rd ed. New York: W.H. Freeman and Company; 1999.

[26] Goldberg A, Schepens S. Measurement error and minimum detectable change in 4-meter gait speed in older adults. Aging Clin Exp Res. 2011;23(5–6):406–12. Epub 2012/04/25. doi: 10.1007/BF03325236 . 22526072

[27] Peters DM, Fritz SL, Krotish DE. Assessing the reliability and validity of a shorter walk test compared with the 10-Meter Walk Test for measurements of gait speed in healthy, older adults. Journal of geriatric physical therapy (2001). 2013;36(1):24–30. Epub 2012/03/15. doi: 10.1519/JPT.0b013e318248e20d . 22415358

[28] Rydwik E, Bergland A, Forsén L, Frändin K. Investigation into the reliability and validity of the measurement of elderly people’s clinical walking speed: a systematic review. Physiother Theory Pract. 2012;28(3):238–56. Epub 2011/09/21. doi: 10.3109/09593985.2011.601804 . 21929322

[29] Middleton A, Fritz SL, Lusardi M. Walking speed: the functional vital sign. Journal of aging and physical activity. 2015;23(2):314–22. Epub 2014/05/09. doi: 10.1123/japa.2013-0236 ; PubMed Central PMCID: PMC4254896. 24812254 PMC4254896

[30] Howell AM, Kobayashi T, Hayes HA, Foreman KB, Bamberg SJ. Kinetic Gait Analysis Using a Low-Cost Insole. IEEE Trans Biomed Eng. 2013;60(12):3284–90. Epub 2013/03/12. doi: 10.1109/TBME.2013.2250972 . 23475336

[31] Saito M, Nakajima K, Takano C, Ohta Y, Sugimoto C, Ezoe R, et al. An in-shoe device to measure plantar pressure during daily human activity. Medical engineering & physics. 2011;33(5):638–45. Epub 2011/02/12. doi: 10.1016/j.medengphy.2011.01.001 . 21310644

[32] Galea MP, Cofré Lizama LE, Butzkueven H, Kilpatrick TJ. Gait and balance deterioration over a 12-month period in multiple sclerosis patients with EDSS scores ≤ 3.0. NeuroRehabilitation. 2017;40(2):277–84. Epub 2017/02/23. doi: 10.3233/nre-161413 . 28222549

[33] Martin CL, Phillips BA, Kilpatrick TJ, Butzkueven H, Tubridy N, McDonald E, et al. Gait and balance impairment in early multiple sclerosis in the absence of clinical disability. Multiple sclerosis (Houndmills, Basingstoke, England). 2006;12(5):620–8. Epub 2006/11/08. doi: 10.1177/1352458506070658 . 17086909

[34] Viqueira Villarejo M, Maeso García J, García Zapirain B, Méndez Zorrilla A. Technological solution for determining gait parameters using pressure sensors: a case study of multiple sclerosis patients. Bio-medical materials and engineering. 2014;24(6):3511–22. Epub 2014/09/18. doi: 10.3233/BME-141177 . 25227064

[35] Mason BS, Rhodes JM, Goosey-Tolfrey VL. Validity and reliability of an inertial sensor for wheelchair court sports performance. J Appl Biomech. 2014;30(2):326–31. Epub 2013/10/23. doi: 10.1123/jab.2013-0148 . 24146035

[36] Tien I, Glaser SD, Aminoff MJ. Characterization of gait abnormalities in Parkinson’s disease using a wireless inertial sensor system. Annual International Conference of the IEEE Engineering in Medicine and Biology Society IEEE Engineering in Medicine and Biology Society Annual International Conference. 2010;2010:3353–6. Epub 2010/11/26. doi: 10.1109/iembs.2010.5627904 . 21097233

