# Supplementary figures and images for: Study for the validation of the FeetMe® integrated sensor insole system compared to GAITRite® system to assess gait characteristics in patients with multiple sclerosis
Source: PLoS One. 2023 Feb 9;18(2):e0272596. doi: 10.1371/journal.pone.0272596 (PMC9910712; doi:10.1371/journal.pone.0272596)

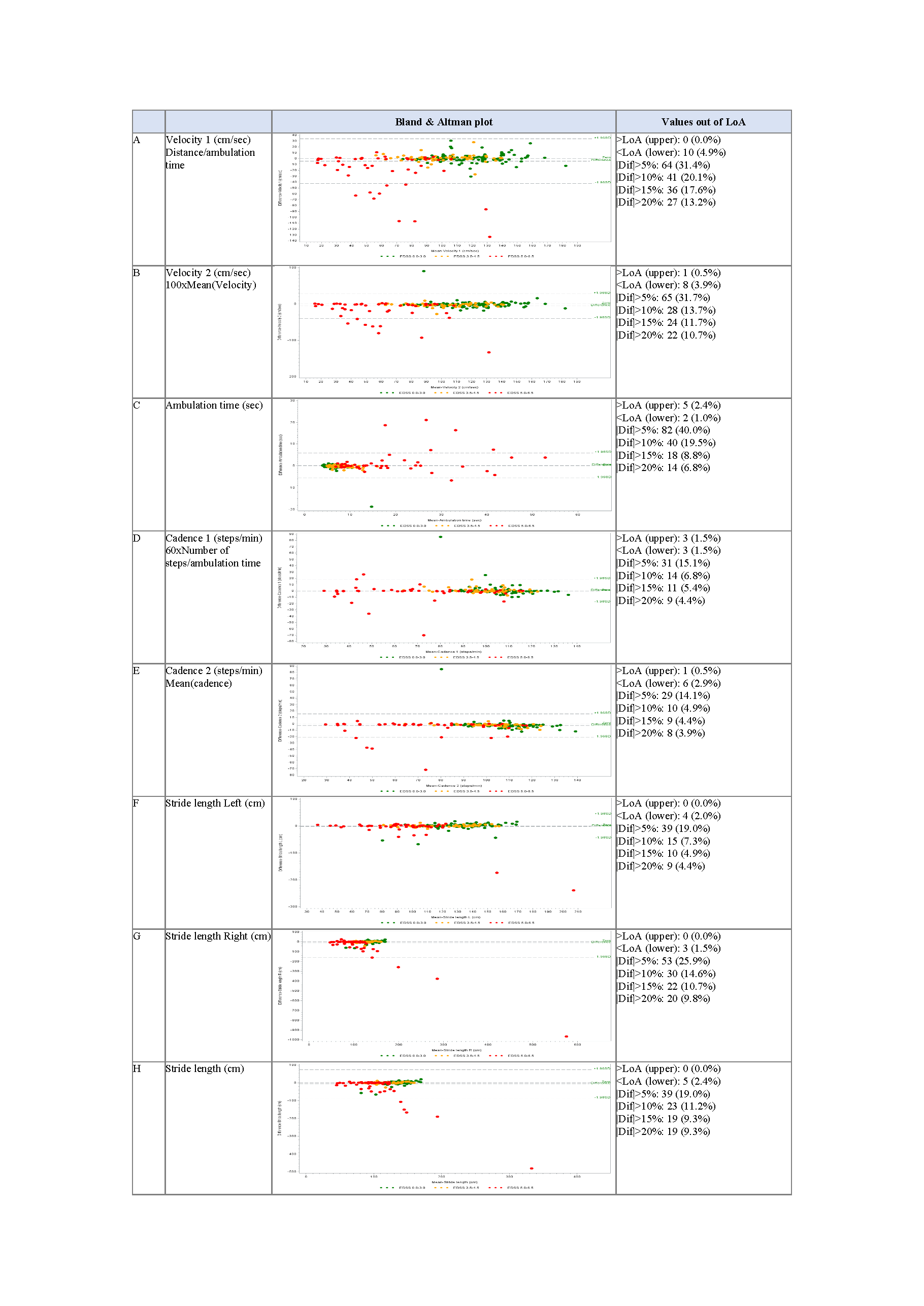

Supplement: S1 Fig — LOA = Limits of Agreement. (TIFF) [file pone.0272596.s001.tiff]
