# Supplementary material for: Study for the validation of the FeetMe® integrated sensor insole system compared to GAITRite® system to assess gait characteristics in patients with multiple sclerosis
Source: PLoS One. 2023 Feb 9;18(2):e0272596. doi: 10.1371/journal.pone.0272596 (PMC9910712; doi:10.1371/journal.pone.0272596)
Supplement: S1 Table — 25SG = T25WT-both devices subject group; 25SG+VD = T25WT-both devices subject group with valid data; ICC = Intra-class correlations; SD = Standard deviation; Criteria for agreement (ICC): 0<ICC<0.3 = Poor; 0.3≤ICC<0.5 = Fair; 0.5≤ICC<0.7 = Moderate; 0.7≤ICC<0.8 = Strong; ICC≥0.8 = Almost perfect. (DOCX) [file pone.0272596.s002.docx]

**S1 Table.** **Agreement between different gait parameters for GAITRite^®^ and FeetMe^®^ Monitor devices within sub-populations.**

|  | **N** | **GAITRite^®^**  **Mean (SD)** | **FeetMe^®^ Monitor** **Mean (SD)** | **Difference**  **Mean (SD)** | **ICC** |
| --- | --- | --- | --- | --- | --- |
| **Velocity 1 (cm/sec)** | | | | | |
| 25SG population | 204 | 98.9 (35.9) | 103.3 (32.3) | -4.4 (19.4) | 0.833 |
| 25SG+VD population | 127 | 104.5 (31.6) | 107.6 (30.3) | -3.2 (14.7) | 0.883 |
| Excluding outliers for GAITRite^®^ and FeetMe^®^ Monitor devices | 197 | 100.8 (33.4) | 104.3 (29.1) | -3.5 (16.9) | 0.849 |
| Excluding values out-of-range for FeetMe^®^ Monitor and GAITRite^®^ device | 204 | 98.9 (35.9) | 103.3 (32.3) | -4.4 (19.4) | 0.8312 |
| Excluding uncertain values (stride length and swing duration) in FeetMe^®^ Monitor device for 25SG population | 204 | 98.9 (35.9) | 103.4 (32.3) | -4.5 (19.4) | 0.833 |
| Excluding all uncertain values in FeetMe^®^ device for 25SG population | 203 | 99.2 (35.7) | 99.3 (34.2) | -0.1 (11.7) | 0.945 |
| **Velocity 2 (cm/sec)** | | | | | |
| Velocity 1 (cm/sec)  Distance/ambulation time | 205 | 98.6 (36.1) | 104.1 (33.2) | -5.5 (17.3) | 0.865 |
| 25SG population | 127 | 104.5 (31.6) | 108.9 (31.0) | -4.5 (13.4) | 0.899 |
| 25SG+VD population | 199 | 100.9 (34.0) | 105.6 (30.6) | -4.7 (14.6) | 0.889 |
| Excluding outliers for GAITRite^®^ and FeetMe^®^ Monitor devices | 205 | 98.6 (36.1) | 104.1 (33.2) | -5.5 (17.3) | 0.865 |
| Excluding values out-of-range for FeetMe^®^ Monitor and GAITRite^®^ device | 205 | 98.6 (36.1) | 104.1(33.2) | -5.5 (17.3) | 0.833 |
| **Ambulation time (sec)** | | | | | |
| Velocity 1 (cm/sec)  Distance/ambulation time | 205 | 9.7 (8.2) | 9.6 (7.5) | 0.1 (2.9) | 0.932 |
| 25SG population | 127 | 8.1 (5.0) | 8.1 (5.1) | -0.01 (0.3) | 0.998 |
| 25SG+VD population | 177 | 7.1 (2.0) | 7.2 (2.1) | -0.1 (0.5) | 0.964 |
| Excluding outliers for GAITRite^®^ and FeetMe^®^ Monitor devices | 205 | 9.7 (8.2) | 9.6 (7.5) | 0.1 (2.9) | 0.932 |
| Excluding values out-of-range for FeetMe^®^ Monitor and GAITRite^®^ device | 205 | 9.7 (8.2) | 9.6 (7.5) | 0.2 (2.9) | 0.932 |
| **Cadence 1 (steps/min)** | | | | | |
| Velocity 1 (cm/sec)  Distance/ambulation time | 205 | 96.7 (21.9) | 96.7 (21.9) | 0.0 (9.4) | 0.908 |
| 25SG population | 127 | 100.4 (17.2) | 100.7 (17.5) | -0.3 (2.7) | 0.988 |
| 25SG+VD population | 179 | 103.0 (12.0) | 102.9 (12.6) | 0.1 (4.1) | 0.945 |
| Excluding outliers for GAITRite^®^ and FeetMe^®^ Monitor devices | 205 | 96.7 (21.9) | 96.7 (21.9) | 0.04 (9.4) | 0.908 |
| Excluding values out-of-range for FeetMe^®^ Monitor and GAITRite^®^ device | 205 | 96.7 (21.9) | 96.7 (21.9) | 0.04 (9.4) | 0.908 |
| **Cadence 2 (steps/min)** | | | | | |
| Velocity 1 (cm/sec)  Distance/ambulation time | 205 | 96.7 (21.9) | 99.5 (21.5) | -2.8 (9.3) | 0.901 |
| 25SG population | 127 | 100.4 (17.1) | 102.9 (17.9) | -2.5 (2.7) | 0.978 |
| 25SG+VD population | 179 | 103.0 (12.0) | 105.6 (12.6) | -2.5 (3.4) | 0.942 |
| Excluding outliers for GAITRite^®^ and FeetMe^®^ Monitor devices | 205 | 96.7 (21.9) | 99.5 (21.5) | -2.8 (9.3) | 0.901 |
| Excluding values out-of-range for FeetMe^®^ Monitor and GAITRite^®^ device | 205 | 96.7 (21.9) | 99.6 (21.4) | -2.8 (9.4) | 0.898 |
| **Stride length Right (cm)** | | | | | |
| Velocity 1 (cm/sec)  Distance/ambulation time | 204 | 119.0 (26.5) | 130.9 (75.5) | -11.9 (76.9) | 0.068 |
| 25SG population | 127 | 122.7 (24.1) | 127.7 (37.9) | -5.0 (36.3) | 0.343 |
| 25SG+VD population | 195 | 121.4 (24.2) | 124.1 (21.9) | -2.7 (15.7) | 0.765 |
| Excluding outliers for GAITRite^®^ and FeetMe^®^ Monitor devices | 201 | 119.5 (26.4) | 123.6 (25.1) | -4.1 (20.1) | 0.686 |
| Excluding values out-of-range for FeetMe^®^ and GAITRite^®^ device | 204 | 119.0 (26.5) | 130.9 (75.5) | -11.8 (76.9) | 0.069 |
| **Stride length Left (cm)** | | | | | |
| Velocity 1 (cm/sec)  Distance/ambulation time | 205 | 118.7 (26.4) | 120.8 (29.4) | -2.1 (22.6) | 0.673 |
| 25SG population | 127 | 122.6 (23.9) | 122.8 (22.1) | -0.3 (9.4) | 0.918 |
| 25SG+VD population | 198 | 120.9 (23.9) | 121.0 (21.7) | -0.1 (9.5) | 0.914 |
| Excluding outliers for GAITRite^®^ and FeetMe^®^ Monitor devices | 204 | 118.9 (26.4) | 119.8 (25.7) | -0.9 (15.7) | 0.827 |
| Excluding values out-of-range  for FeetMe^®^ Monitor and GAITRite^®^ device | 205 | 118.7 (26.4) | 120.8 (29.4) | -2.1 (22.6) | 0.673 |
| **Stride length (cm)** | | | | | |
| Velocity 1 (cm/sec)  Distance/ambulation time | 204 | 119.0 (26.4) | 126.0 (42.1) | -7.0 (41.2) | 0.301 |
| 25SG population | 127 | 122.6 (24.0) | 125.3 (25.6) | -2.6 (19.8) | 0.679 |
| 25SG+VD population | 193 | 122.0 (23.3) | 123.6 (20.0) | -1.6 (11.5) | 0.858 |
| Excluding outliers for GAITRite^®^ and FeetMe^®^ devices | 203 | 119.1 (26.4) | 123.8 (28.0) | -4.7 (24.3) | 0.589 |
| Excluding values out-of-range for FeetMe^®^ and GAITRite^®^ device | 204 | 119.0 (26.4) | 126.0 (42.1) | -7.0 (41.2) | 0.301 |

25SG=T25WT-both devices subject group; 25SG+VD=T25WT-both devices subject group with valid data; ICC=Intra-class correlations; SD=Standard deviation; Criteria for agreement (ICC): 0<ICC<0.3=Poor; 0.3≤ICC<0.5=Fair; 0.5≤ICC<0.7=Moderate; 0.7≤ICC<0.8 = Strong; ICC≥0.8=Almost perfect.
